# Impact of vitamin D levels on mortality in older covid-19 vaccinated patients

**DOI:** 10.1186/s12877-025-05873-1

**Published:** 2025-04-10

**Authors:** Chiara Ceolin, Margherita Vergadoro, Cristina Simonato, Sara Cazzavillan, Mario Virgilio Papa, Giulia Salerno Trapella, Benedetta Di Marzio, Riccardo Sermasi, Bruno Micael Zanforlini, Chiara Curreri, Anna Bertocco, Maria Devita, Alessandra Coin, Luca Spiezia, Giuseppe Sergi, Marina De Rui

**Affiliations:** 1 Department of Medicine (DIMED) Geriatrics Division, University of Padua, Via Giustiniani 2, Padua, 35128 Italy; 2 Department of Medicine, First Chair of Internal Medicine, Padova University Hospital, Padua, Italy; 3 Department of Women’s and Children’s Health, School of Community Medicine and Primary Health Care, University of Padua, Padua, Italy; 4 Department of General Psychology (DPG), University of Padua, Padua, Italy

**Keywords:** Vitamin D, Vaccination, COVID-19, SARS-CoV-2, Older adults

## Abstract

**Background:**

Vitamin D plays a key role in regulating the immune system and vaccine response, and hypovitaminosis D is a known risk factor for mortality. However, its potential influence on mortality in SARS-CoV-2 vaccinated older adults remains underexplored. This study aims to examine survival differences between unvaccinated and vaccinated older adults with varying vitamin D levels, and to assess the impact of vitamin D on mortality.

**Methods:**

We recruited patients aged 65 and over from the Geriatrics Unit of Azienda Ospedale - Università Padova. Clinical, pharmacological data, including vaccination status and vitamin D levels, were collected at admission, alongside mortality data 12 months post-hospitalization. Participants were divided into three groups: unvaccinated, vaccinated with vitamin D levels of 25–50 nmol/L, and vaccinated with levels > 50 nmol/L.

**Results:**

A total of 126 participants were included (56% women, mean age 83 years). No significant differences were found in COVID-19 severity among the three groups. After 12 months, 24 deaths were recorded: 17% in unvaccinated, 19% in vaccinated with low vitamin D, and 20% in vaccinated with high vitamin D (*p* = 0.94). Kaplan-Meier curves showed that mortality risk for vaccinated individuals with low vitamin D was similar to unvaccinated patients but significantly higher than vaccinated individuals with high vitamin D (*p* = 0.04). Vitamin D levels of 25–50 nmol/L were associated with a threefold increased risk of 12-month mortality (HR: 3.79, *p* < 0.001).

**Conclusions:**

Vitamin D levels can impact mortality in older vaccinated individuals. Early correction of vitamin D deficiency could potentially enhance outcomes.

**Supplementary Information:**

The online version contains supplementary material available at 10.1186/s12877-025-05873-1.

## Background

The Coronavirus Disease 2019 (COVID-19) pandemic, caused by the severe respiratory disease linked to SARS-CoV-2 infection [1], has profoundly affected the health of older adults [2]. Age-related factors, such as the aging immune system, increased comorbidities, and physiological changes, have rendered old people the most vulnerable victims of this pandemic, with significantly high mortality rates reported within this population [3]. As individuals age, they experience a decline in immune function, characterized by reduced T cell efficacy and elevated inflammatory cytokines—collectively termed immunosenescence—which can exacerbate the severity of COVID-19 infections [4, 5]. Therefore, it is crucial to identify tailored and effective preventive strategies within this demographic to improve health outcomes and overall well-being.

Vitamin D has emerged as a vital nutrient in supporting immune function [6]. This fat-soluble vitamin enhances the pathogen-fighting abilities of immune cells, including macrophages and T cells, which are essential for orchestrating an effective response to infections [7, 8]. Moreover, vitamin D boosts serum levels of human cathelicidin LL-37, a key antimicrobial peptide of the innate immune system [9]. Additionally, vitamin D possesses anti-inflammatory properties that may help mitigate the cytokine storms often observed in severe cases of COVID-19 [10]. Adequate levels of vitamin D have not only been shown to strengthen general immune responses but also to improve vaccine efficacy. Building on the promising findings from vaccination studies—such as those related to influenza—in older populations, a growing body of research indicates that vitamin D may also significantly influence the efficacy of COVID-19 vaccines [11]. Although previous studies have yielded conflicting results regarding the impact of optimal vitamin D levels on antibody responses in SARS-CoV-2 vaccinated individuals, there is evidence supporting the beneficial effect of supplementation on vaccination responses and mortality [12]. However, much of this research has primarily focused on younger patients, leaving a gap in understanding the impact of serum vitamin D levels on vaccine efficacy and mortality among older adults.

We hypothesize that, given the critical role of vitamin D in reducing inflammation and infection risk to optimal levels, vitamin D deficiency—especially among older individuals—may be associated with adverse mortality outcomes. In other words, we expect that vitamin D levels in vaccinated patients may influence survival outcomes in older adults. Therefore, the objective of this study is to examine how survival rates differ between unvaccinated and vaccinated individuals (with varying serum vitamin D levels) within a cohort of older patients eligible for SARS-CoV-2 vaccination. A secondary objective is to explore whether specific vitamin D levels may be associated with 12-month mortality in vaccinated patients.

## Methods

### Study population

The characteristics of the study population have been described in previous reports [2]. In summary, this study involved a consecutive series of Caucasian patients over 65 years old, recruited at the Geriatrics Unit of the Azienda Ospedale - Università Padova, irrespective of their reason for admission, with documented infection of SARS-CoV-2. Patients were excluded if they had a fever, severe dehydration, or heart failure with marked body edema. Furthermore, individuals with advanced dementia who were unable to follow instructions were not included in the study.

The study followed good clinical practice guidelines and adhered to the ethical principles of the Declaration of Helsinki (revised in 2000). The study protocol was approved by the local Ethics Committee (Comitato Etico per la Sperimentazione Clinica della Provincia di Padova, protocol number 16412/AO/23). All participants were fully informed about the risks and benefits of the study, and each provided both verbal and written consent for the publication of the data.

### Data collection

- *Patient Clinical and Pharmacological Characteristics*. The clinical and pharmacological characteristics of the patients, including vaccination status, type of vaccine administered, and the total number of doses received, were collected from medical records by experienced physicians. Comprehensive details on comorbidities, functional autonomy, nutritional status, and anthropometric measurements, as well as COVID-19-related information (such as vaccine doses and associated symptoms), have been previously reported [2].
- *Sarcopenia assessment*. Likewise, the methodologies for assessing muscle strength and body composition, including bioelectrical impedance analysis and sarcopenia diagnosis, are documented in the same reference [2]. Briefly, sarcopenia was diagnosed according to the 2019 European consensus criteria based on muscle strength and mass values [13]. Upper limb strength was evaluated using DynEx electronic hand dynamometers (MD Systems, Westerville, OH, USA) by trained medical personnel. Muscle mass was estimated using bioelectrical impedance analysis with the equation developed by Sergi et al. [14] to calculate appendicular skeletal muscle mass (ASMM). The ASMM index (ASMMI) was obtained by dividing the ASMM by the subject’s height squared.
- *Medication Data*. In addition, data on the total number of medications, with specific attention to vitamin D supplementation, were collected.
- *Vitamin D Levels and Laboratory Analyses*. Serum 25-hydroxy-vitamin D (25-OH-D) levels were measured from blood samples taken at hospital admission, with laboratory analyses performed following standardized protocols at the Laboratory Medicine Unit of the University Hospital of Padua. For the categorization of vitamin D levels, we used the following cutoff values: patients with vitamin D levels < 25 nmol/L were classified as deficient, those with levels between 25 and 50 nmol/L as insufficient, and those with levels > 50 nmol/L as sufficient, according to the international guidelines [15]. Vitamin D levels were measured from blood samples using an immunochemiluminescence assay.
- *Follow-up evaluation*. Mortality data were recorded 12 months (T12) after discharge.

### Statistical analysis

The characteristics of the sample are presented as means ± standard deviations for continuous variables with normal distributions, and as medians with interquartile ranges for those with non-normal distributions. The normality of continuous variables was assessed using the Shapiro-Wilk test. Categorical variables are reported as counts and percentages. Due to the limited number of patients with very low vitamin D levels (< 25 nmol/L), participants were categorized into three groups based on their vaccination status and vitamin D levels: unvaccinated (*n* = 35), vaccinated with vitamin D levels between 25 and 50 nmol/L (*n* = 36), and vaccinated with vitamin D levels above 50 nmol/L (*n* = 55).

The characteristics of these groups were compared using ANOVA for continuous variables and the Chi-square test for categorical variables, depending on the type of data. For survival analysis, Kaplan-Meier curves were generated for the three groups (unvaccinated, and the two vaccinated groups). Additionally, a Cox proportional hazards regression model was used to evaluate whether vitamin D status was an independent predictor of 12-month post-discharge mortality in vaccinated participants. Two models were constructed: the first adjusted for gender and age, and the second further adjusted for additional variables, including MPI, sarcopenia, and length of hospital stay.

Statistical significance was set at *p* < 0.05 for all tests. All analyses were performed using IBM SPSS Statistics, version 29.0 (IBM Corp., Armonk, NY, USA).

## Results

From the initial sample of 192 patients, we excluded individuals with missing data on sarcopenia and vitamin D levels (*n* = 59), as well as those receiving vitamin D supplementation prior to hospitalization (*n* = 7), resulting in a final sample of 126 participants. Table 1 presents the baseline characteristics of the sample stratified by vaccination status and vitamin D levels. Vaccinated participants with vitamin D levels between 25 and 50 nmol/L had higher comorbidity scores and were on more medications at admission compared to the other groups. No significant differences were observed in terms of COVID-19 severity. However, the length of hospital stay was significantly longer in vaccinated individuals with lower vitamin D levels (20 [14; 35] days) compared to those with higher levels (16 [10; 24] days and 15 [10; 23] days, *p* = 0.03). Additionally, hospitalizations due to COVID-19 were more frequent among participants with higher vitamin D levels (53% vs. 21% vs. 39%, *p* = 0.01). No further differences were observed, even when considering readmissions during the follow-up period. In terms of the multidimensional geriatric assessment, participants with low vitamin D levels showed lower ADL scores (1 [1; 2] vs. 1 [1; 6] and 2 [1; 6], *p* = 0.03). Finally, sarcopenia was more prevalent in vaccinated individuals with low vitamin D levels (50% vs. 28% vs. 19%). Among vaccinated patients, 71% of the sample had received at least one dose of an mRNA vaccine. Among them, 85 patients had received a second dose, while only 27 had completed the third dose (data not shown). When considering gender differences, no significant variations in vitamin D levels were observed. However, women exhibited greater impairments in Activities of Daily Living (ADL) and total Multidimensional Prognostic Index (MPI) values, while men demonstrated a higher prevalence of sarcopenia (44% vs. 19%, *p* = 0.003-data not shown).

**Table 1:** Characteristics of the sample at baseline according to vaccinal status and presence of sarcopenia

Variable	All (*n* = 126)	Not vaccinated (*n* = 35)	Vaccinated (*n* = 91)		*p*-value
			Vitamin D levels 25–50 nmol/L (*n* = 36)	Vitamin D levels > 50 nmol/L (*n* = 55)	
**Age [years]**	83 ± 7	81 ± 8	83 ± 7	83 ± 6	0.42
**Gender F**	70 (56%)	20 (57%)	20 (56%)	30 (55%)	0.97
**CIRS-CI**	3 [2;5]	2 [1;3]	3.5 [3;6]	3 [2;5]	***0.002***
**No. of drugs at admission**	5 [3.25;8]	3 [0.5;6]	7 [5;9]	6 [4;8]	***< 0.001***
**Smoking habits [%]**					0.44
Active	8 (7%)	0	3 (9%)	5 (10%)	
Previous	26 (22%)	9 (27%)	7 (21%)	10 (20%)	
**COVID-19 severity**					
O_2_ at admission [L/min]	0 [0;4]	2 [0;6]	1 [0;4]	0 [0;2]	0.10
Pneumonia at admission [%]	68 (57%)	22 (65%)	21 (58%)	25 (51%)	0.46
Length of stay [days]	16 [11;25]	16 [10;24]	20 [14;35]	15 [10;23]	***0.03***
Intensive Care [%]	7 (6%)	1 (3%)	4 (13%)	2 (4%)	0.20
Hospitalization unrelated to COVID [%]	49 (40%)	7 (21%)	14 (39%)	28 (53%)	***0.01***
**Multidimensional geriatric assessment**					
ADL	1 [1;5]	1 [1;6]	1 [1;2]	2 [1;6]	***0.03***
MNA	18 ± 5	18 ± 5	17 ± 4	19 ± 4	0.13
MPI	0.6 [0.4;0.8]	0.6 [0.3;0.8]	0.7 [0. 6;0.8]	0.5 [0.4;0.7]	0.06
**Body composition**					
ASMMI [Kg/m^2^]	6 ± 1	6 ± 1	6 ± 1	6 ± 1	0.96
BMI [Kg/m^2^]	27 ± 7	26 ± 6	28 ± 7	27 ± 7	0.56
MHG [Kg_f_]	18 [12;25]	17 [9;29]	14 [11;20]	20 [12;25]	0.13
Sarcopenic people	30 (23.8%)	8 (22.8%)	14 (38.9%)	8 (14.5%)	***0.01***

*Notes*: Values are expressed as means ± standard deviation, medians (interquartile range) or counts (percentages %) as appropriate

*Abbreviations*: F = females; CIRS-CI = Cumulative Illness Rating Scale - Comorbidity Index; O_2_ = Oxygen; ADL = Activities of Daily Living; MNA = Mini Nutritional Assessment; MPI = Multidimensional Prognostic Index; ASMMI = Appendicular Muscle Mass Index; BMI = Body Mass Index; MHG = Maximum Handgrip Strength. P-values < 0.05 are reported in bold

**Fig. 1:**
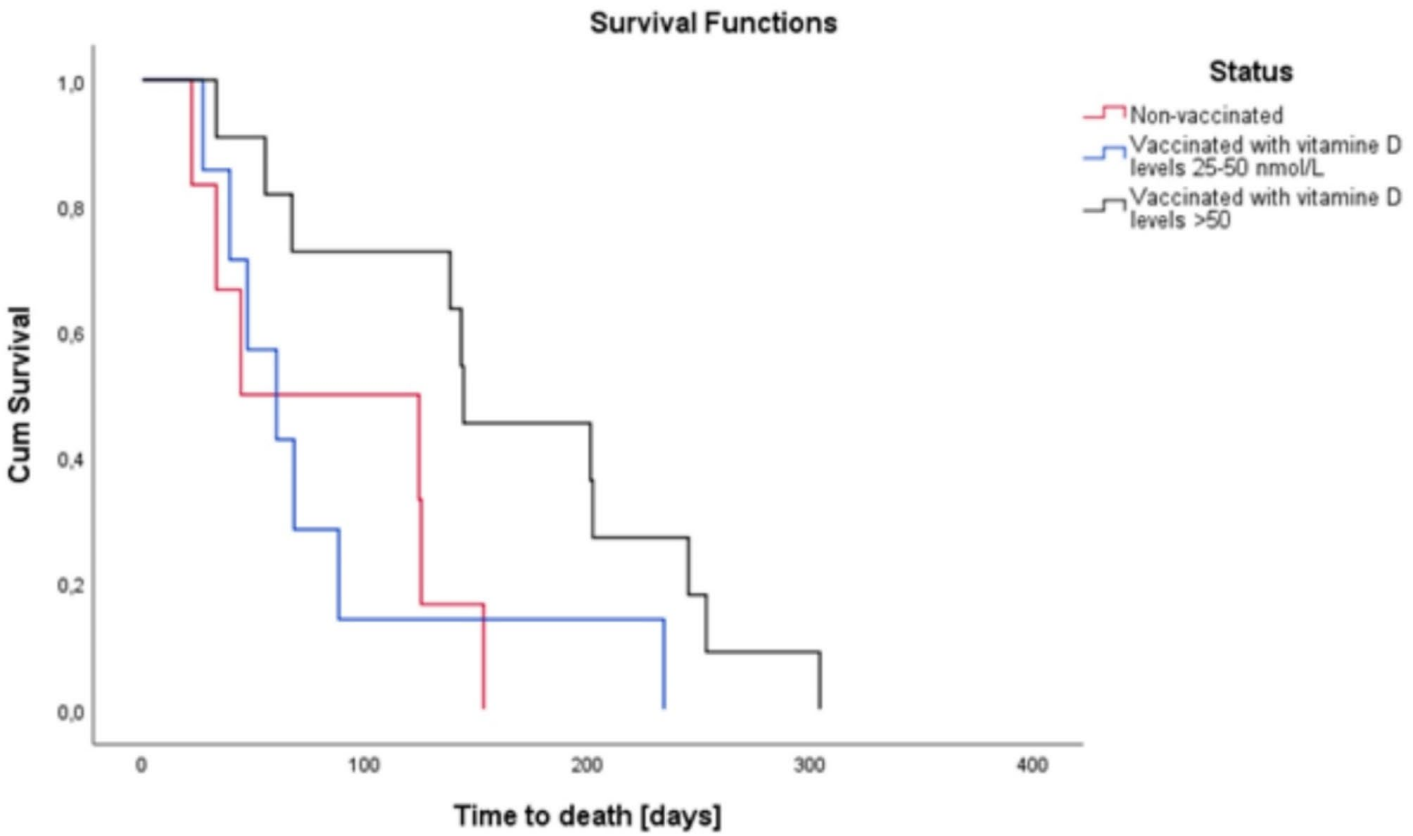
Kaplan-Meyer survival curves: mortality at 12 months after discharge according to vaccinal status and vitamin D levels

After 12 months of follow-up, a total of 24 deaths were recorded: 6 (17%) among unvaccinated participants, 7 (19%) among vaccinated individuals with low vitamin D levels, and 11 (20%) among those with high vitamin D levels (*p* = 0.94). No significant differences in mortality rates were observed based on sex. The Kaplan-Meier survival curves (Fig. 1) demonstrate that the initial mortality risk for unvaccinated patients was comparable to that of vaccinated individuals with low vitamin D levels. However, over time, vaccinated patients with low vitamin D levels exhibited a worse survival rate, particularly in comparison to vaccinated participants with high vitamin D levels (*p* = 0.04). This significance approached statistical relevance when analyzed separately by sex (women: *p* = 0.07; men: *p* = 0.08).

Table 2 presents the results of the Cox regression analysis conducted among vaccinated patients. In Model 1, vitamin D levels were identified as a significant risk factor for mortality. Patients with vitamin D levels between 25 and 50 nmol/L were nearly three times more likely to experience mortality within 12 months compared to those with higher vitamin D levels (> 50 nmol/L) (HR 2.91, *p* = 0.02). In Model 2, both the presence of sarcopenia and vitamin D levels between 25 and 50 nmol/L were strongly associated with increased mortality, with hazard ratios of 3.81 (*p* = 0.03) and 4.52 (*p* = 0.04), respectively. No significant associations were found for sex, age, or MPI scores.

**Table 2:** Cox regression analysis of one-year post-discharge mortality in vaccinated patients

Model	Outcome	Variable	HR	*p-value*	IC 95%	
					Lower limit	Upper limit
**1**	*Mortality per 1 point* *increase in variable or per specified category*	Age [years]	1	0.66	0.94	1.04
		Gender F	1.47	0.32	0.68	3.17
		Vitamin D levels				
		25–50 nmol/L	**2.91**	**0.02**	**1.51**	**7.37**
		> 50 nmol/L	0.47	0.17	0.21	1.06
**2**	*Mortality per 1 point* *increase in variable or per specified category*	Age [years]	0.9	0.07	0.8	1.01
		Gender F	1.59	0.45	0.47	5.38
		MPI	3.94	0.13	0.38	4.17
		Length of stay	1.02	0.51	0.97	1.06
		Sarcopenia	**3.81**	**0.03**	**2.1**	**4.5**
		Vitamin D levels				
		25–50 nmol/L	**4.52**	**0.04**	**1.04**	**7.73**
		> 50 nmol/L	0.43	0.19	0.12	1.51

*Abbreviations*: HR = Hazard Ratio; F = Female; MPI: Multidimensional Prognostic Index Model 1 was adjusted for gender, and age. Model 2 was adjusted for gender, age, MPI, length of stay, and presence of sarcopenia. P-values < 0.05 are reported in bold

## Discussion

This study focused on the impact of vitamin D deficiency on mortality in old patients vaccinated against COVID-19. Two key findings emerged from the analysis: (1) Being vaccinated but having low vitamin D levels is associated with a survival rate similar to that of unvaccinated individuals, and significantly lower than that of vaccinated individuals with vitamin D levels above 50 nmol/L. (2) Vitamin D levels between 25 and 50 nmol/L are a risk factor for 12-month mortality in vaccinated patients, associated with an approximately threefold increased risk of death. These results highlight the association between vitamin D levels and mortality in the older population, suggesting that low vitamin D levels, regardless of vaccination status, represent an important factor associated with mortality.

Vitamin D is well-known for its role in immune regulation, influencing both innate and adaptive responses [16]. The active form, 1,25-dihydroxyvitamin D (1,25[OH]2D), suppresses pro-inflammatory cytokine production in monocytes and macrophages by modulating MAP kinase phosphatase 1 [17], regulates the mTOR pathway [18], and enhances T cell activation by inducing phospholipase C-gamma 1 [19]. Beyond its role in immune modulation, vitamin D supplementation enhances vaccine responses, particularly in older adults, improving immunity against viruses like varicella zoster, influenza, rubella, and hepatitis B [20–22]. However, some studies in old populations report conflicting results regarding its impact on vaccine-induced immunity [23]. Given these observations, the potential role of vitamin D in modulating immune responses to SARS-CoV-2 vaccination has become a topic of interest. Vitamin D’s antiviral effects are believed to be driven by its induction of antimicrobial peptides, which reduce viral replication and suppress excessive inflammatory responses that can lead to conditions like Acute Respiratory Distress Syndrome (ARDS) in severe COVID-19 cases [24, 25]. Additionally, vitamin D influences the renin-angiotensin system and has anti-thrombotic effects, which are critical in the context of COVID-19, and may help resolve the harmful pro-inflammatory responses seen in severe cases [24–26]. Supporting the importance of vitamin D in COVID-19 outcomes, a 2021 meta-analysis of 54 studies encompassing over 1.4 million individuals highlighted that patients with severe vitamin D deficiency were at greater risk for ARDS, Intensive Care Unit admission, and mortality due to COVID-19, as well as a higher likelihood of infection and hospitalization [27]. In contrast, our study did not reveal significant differences among patient groups with varying vitamin D levels regarding COVID-19 severity or hospitalization rates. One notable finding was that patients with low vitamin D levels experienced a longer hospital stay, which may serve as an indicator of poor overall health status [28]. However, whether vitamin D supplementation can enhance the immunogenicity or efficacy of SARS-CoV-2 vaccines remains an open question [28, 29]. Observational studies have reported conflicting results: some suggest that individuals with higher circulating 25(OH)D levels or those taking vitamin D supplements show stronger post-vaccination antibody responses [30, 31], while others find no significant association [32, 33]. Recently, Di Filippo et al. noted that low baseline 25(OH)D negatively impacted long-term antibody responses in healthcare workers [34]. A significant limitation of these studies is their primary focus on younger individuals, with insufficient attention given to older adults. This is particularly concerning, as the old people may exhibit distinct immunological profiles and are more vulnerable to severe COVID-19 outcomes. The lack of research in this population creates significant gaps in understanding how interventions, such as vitamin D supplementation, might influence vaccine responses and overall immunity. In this study, although we did not measure T-cells or antibody levels in the patients’ blood, we focused on comparing vaccinated and unvaccinated individuals to investigate the relationship between vitamin D levels and 12-month mortality outcomes. The survival curves for vaccinated participants with low vitamin D levels were comparable to those of unvaccinated individuals, while vaccinated participants with higher vitamin D levels showed a distinct advantage over both groups. Our findings suggest that adequate vitamin D levels– especially above 50 nmol/L– may be associated with better mortality outcomes in older adults, although the underlying mechanisms remain to be fully explored.

Over the past few decades, large cohort studies have shown that vitamin D deficiency raises the risk of all-cause mortality, while regular supplementation reduces mortality across various conditions [35]. Meta-analyses of randomized controlled trials confirm the benefits of addressing vitamin D deficiency, particularly in those with low levels. However, the impact of supplementation in individuals with sufficient vitamin D remains unclear, suggesting that the benefits may be more pronounced in deficient populations [11, 36]. Recent studies and meta-analyses of randomized controlled trials (RCTs) examining the relationship between vitamin D and mortality have not consistently demonstrated a causal link between serum vitamin D levels and all-cause mortality [37, 38]. In many cases, the association was not statistically significant, which may be partly due to the limited representation of participants with 25(OH)D concentrations below 20 ng/mL in RCTs [39, 40]. In the context of COVID-19 mortality, severe vitamin D deficiency in patients with acute respiratory failure due to COVID-19 has been identified as a key predictor of 10-day mortality [41]. Similarly, various systematic reviews have pointed to vitamin D status as a determinant of infection risk, disease severity, and COVID-19-related mortality [42, 43]. These findings suggest that low vitamin D levels may be more of a predictive factor than a mere consequence of the infection. In the present study, our goal was not to establish a definitive causal relationship between vitamin D and mortality, but rather to examine the association between these two variables within an older cohort. Our results indicate that vitamin D levels between 25 and 50 nmol/L are significantly associated with an increased risk of 12-month all-cause mortality in vaccinated individuals, even after adjusting for potential confounding factors. These findings contribute to the growing body of evidence linking vitamin D status to health outcomes, particularly in older adults. While the precise mechanism underlying this association remains unclear, it is more likely that low vitamin D levels contribute to poor health status, which in turn is consistently linked to more unfavorable outcomes. In the context of vaccination, our study suggests that vitamin D levels may influence the effectiveness of the immune response in older adults, with those having lower levels showing outcomes similar to unvaccinated individuals. These results highlight the need for considering vitamin D status as part of broader health management strategies, especially for older populations, and emphasize the importance of optimizing vitamin D levels as a complementary factor in improving long-term health outcomes, particularly in conjunction with vaccination.

Some limitations must be acknowledged. Foremost among them is the small sample size, which reduces statistical power and limits our ability to explore potential gender differences. Moreover, this study did not assess immune response markers, such as T-cell activity, and therefore did not provide direct evidence of how vitamin D impacts the immune response in the vaccinated cohort. On the other hand, a significant strength of this study is its focus on a cohort of exclusively older patients, who were assessed 12 months after hospitalization, with nearly complete data on vaccination status.

## Concusions

Vitamin D levels are associated with health outcomes in older patients vaccinated against SARS-CoV-2. While these findings suggest that timely correction of vitamin D deficiency may contribute to improved survival, further research is necessary to validate these results. Future studies should explore the effect of vitamin D on the immune system, identify or propose optimal thresholds for vitamin D levels associated with better health outcomes, and investigate potential gender differences.

## Electronic supplementary material

Below is the link to the electronic supplementary material.

Supplementary Material 1

## Data Availability

The data presented in this study are available on request from the corresponding author due to privacy restrictions.
