# Supplementary material for: Impact of vitamin D levels on mortality in older covid-19 vaccinated patients
Source: BMC Geriatr. 2025 Apr 10;25:240. doi: 10.1186/s12877-025-05873-1 (PMC11983984; doi:10.1186/s12877-025-05873-1)
Supplement: Supplementary file 1 — Supplementary Material 1 [file 12877_2025_5873_MOESM1_ESM.docx]

**Kaplan-Meyer table**:

| **Group** | **Time (days)** | **N° at risck** | **Cumulative eventis** | **Cumulative probability of survival** | **Standard Error** | **N° remaining** |
| --- | --- | --- | --- | --- | --- | --- |
| Unvaccinated patients | 22 | 6 | 1 | 0.833 | 0.152 | 5 |
| Unvaccinated patients | 33 | 5 | 2 | 0.667 | 0.192 | 4 |
| Unvaccinated patients | 44 | 4 | 3 | 0.5 | 0.204 | 3 |
| Unvaccinated patients | 124 | 3 | 4 | 0.333 | 0.192 | 2 |
| Unvaccinated patients | 125 | 2 | 5 | 0.167 | 0.152 | 1 |
| Unvaccinated patients | 153 | 1 | 6 | 0 | 0 | 0 |
| Vaccinated with vitamin D levels 25-50 nmol/L | 27 | 7 | 1 | 0.857 | 0.132 | 6 |
| Vaccinated with vitamin D levels 25-50 nmol/L | 39 | 6 | 2 | 0.714 | 0.171 | 5 |
| Vaccinated with vitamin D levels 25-50 nmol/L | 47 | 5 | 3 | 0.571 | 0.187 | 4 |
| Vaccinated with vitamin D levels 25-50 nmol/L | 60 | 4 | 4 | 0.429 | 0.187 | 3 |
| Vaccinated with vitamin D levels 25-50 nmol/L | 68 | 3 | 5 | 0.286 | 0.171 | 2 |
| Vaccinated with vitamin D levels 25-50 nmol/L | 88 | 2 | 6 | 0.143 | 0.132 | 1 |
| Vaccinated with vitamin D levels 25-50 nmol/L | 234 | 1 | 7 | 0 | 0 | 0 |
| Vaccinated with vitamin D levels >50 nmol/L | 33 | 11 | 1 | 0.909 | 0.087 | 10 |
| Vaccinated with vitamin D levels >50 nmol/L | 55 | 10 | 2 | 0.818 | 0.116 | 9 |
| Vaccinated with vitamin D levels >50 nmol/L | 67 | 9 | 3 | 0.727 | 0.134 | 8 |
| Vaccinated with vitamin D levels >50 nmol/L | 138 | 8 | 4 | 0.636 | 0.145 | 7 |
| Vaccinated with vitamin D levels >50 nmol/L | 143 | 7 | 5 | 0.545 | 0.15 | 6 |
| Vaccinated with vitamin D levels >50 nmol/L | 144 | 6 | 6 | 0.455 | 0.15 | 5 |
| Vaccinated with vitamin D levels >50 nmol/L | 201 | 5 | 7 | 0.364 | 0.145 | 4 |
| Vaccinated with vitamin D levels >50 nmol/L | 202 | 4 | 8 | 0.273 | 0.134 | 3 |
| Vaccinated with vitamin D levels >50 nmol/L | 245 | 3 | 9 | 0.182 | 0.116 | 2 |
| Vaccinated with vitamin D levels >50 nmol/L | 253 | 2 | 10 | 0.091 | 0.087 | 1 |
| Vaccinated with vitamin D levels >50 nmol/L | 304 | 1 | 11 | 0 | 0 | 0 |
